# Gut Check: The evolution of an educational board game

**DOI:** 10.1371/journal.pbio.2001984

**Published:** 2017-04-28

**Authors:** David A. Coil, Cassandra L. Ettinger, Jonathan A. Eisen

**Affiliations:** 1 Genome Center, University of California, Davis, Davis, California, United States of America; 2 Evolution and Ecology, Medical Microbiology and Immunology, University of California, Davis, Davis, California, United States of America

## Abstract

The “gamification” of science has gained a lot of traction in recent years, and games that convey scientific concepts or themes are increasingly popular. While a number of existing games touch on microbiology, very few consider the beneficial (as opposed to the detrimental) aspects of microbes. We designed a board game called “Gut Check: The Microbiome Game” to fill this gap. The game is meant to be both educational as well as challenging and fun. Here we discuss the development of the game, some of the logistics of game development in this context, and offer suggestions for others thinking of similar projects.

## Background

Making science more accessible and interesting to the public through the process of “gamification” has become increasingly popular in recent years [1]. Gamification is loosely defined as the application of game principles and game design in a nongame context [2]. This approach has been particularly popular in citizen science, in which the public is engaged in the collection and/or analysis of data. Witness the success of projects such as Foldit [3] and EyeWire [4], which have thousands of dedicated followers or “players” who help solve protein structures and map neurons, respectively. A related concept is called “game-based learning” or more commonly, “educational games.” Long popular with teachers as an active learning approach, and more recently popular in medicine [5], educational games increase engagement with subject material through playing a game. Probably the most well-known example in schools is the computer game “Oregon Trail” [6], which has been teaching kids since 1971 about the perils of pioneer life.

Recently, “gamification” has also been applied to get players to interact with real-life biological systems, a concept known as “biotic games” [7–9]. These games are overlaid onto biological systems using a computer, and players then manipulate living organisms in a video game–like fashion. These “biotic games” have great potential for classroom learning but are currently limited by either technology or logistical constraints of the biological system under study.

Overall, the vast majority of educational literature on games relates only to video or computer games (e.g., [10–13]). Noncomputer educational games (e.g., card games, dice games, board games, etc.) are also abundant but seem to be much less popular or well known. In our (anecdotal) experience, however, many of these games are not particularly challenging or fun to play. While from a student perspective, playing a lackluster game might be more engaging than a lecture, it seems that a game people would play outside of a classroom or work setting would be more successful.

We think it is important to make a distinction between science-based games (those that use scientific concepts or ideas as part of their theme or mechanics) and science pedagogy games (those designed with pedagogy as the primary goal). Examples of science-based games include *Timeline* [14], which asks players to place inventions or discoveries into an ordered timeline, and *Evolution* [15], which allows players to compete using evolutionary principles (to varying degrees of accuracy). Science pedagogy games and their education focus include *Voyager*: *Satellites* [16] (scientific satellites), *Control-Alt-Hack* [17] (computer security), *ChemMend* [18] (the periodic table), the Go Fish–inspired *Go Extinct*! [19] (interpreting evolutionary trees), *KEEP COOL* [20] (climate change) and *Parasites Unleashed* [21] (parasitic life cycles). The science pedagogy games are often less well known, possibly resulting from limited distribution and the main focus not being on game mechanics, which could limit engagement and/or replayability.

## Gut Check: The Microbiome Game

Microbiology is the study of “microbes”—organisms that are invisible to the naked eye. Most microbe themed games (educational or otherwise) focus on pathogens (“bad” microbes) and what can be viewed as the negative aspects of microbiology. From spreading the plague, to zombie-inducing viruses, to global pandemics, microbes are a popular antagonist. While this approach meshes with a common negative perception of microbes, it does not accurately represent the fact that microbes can have beneficial effects on other organisms. Beneficial microbes play critical (but largely underappreciated) roles in diverse areas from human health to agriculture to the global carbon cycle to animal behavior.

**Fig 1:**
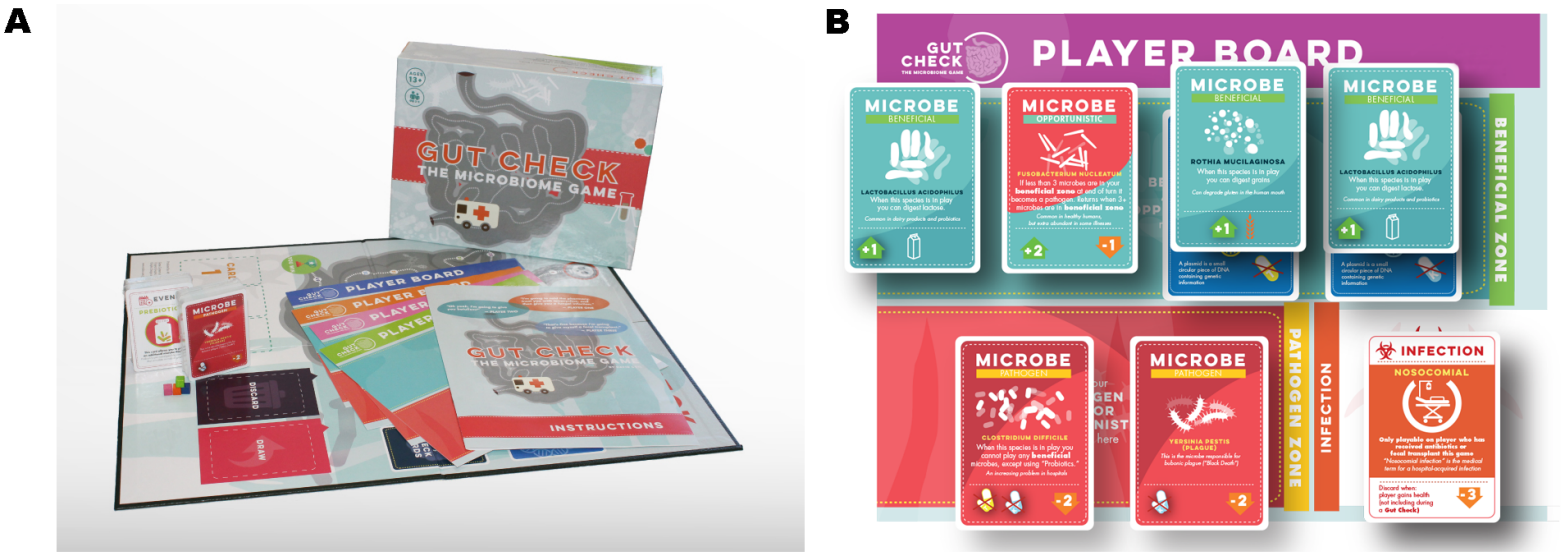
Gut Check: The Microbiome Game (commercial version). (A) Promotional photo of the game showing the game board, instructions, player boards, cards, and box. (B) An example player board set up to have a microbiome containing both beneficial (teal), opportunistic (red and teal), and pathogenic (red) bacteria cards, as well as a unspecified nosocomial infection (orange) card in the lower right. Antibiotic resistance cards (blue) can be seen beneath two of the beneficial bacteria cards.

Our goal was to create a science pedagogy game that would be challenging and interesting to play, require strategic thinking, and also educate people about nonpathogenic microbes. Our intended target audience was high school students and undergraduates with an interest in biology (though in fact the game has been popular with children as young as six). We chose to focus on the human microbiome—the collection of microbes that live in and on people, including the dangerous, the beneficial, and everything in between. The result is Gut Check: The Microbiome Game (Fig 1), available in both a free print-at-home version [22] and a commercial version from MO BIO Laboratories, a QIAGEN company [23]. Both versions of the game are completely open access and are distributed under a CC-BY license. This license allows a product to be modified, redistributed, and even sold commercially by others, as long as the original creators are attributed. It was important to us that the game be as widely available as possible, and we were fortunate to be able to work with both artists and a company who were willing to use an open license. When we began development of our board game, there were a few board games that used microbiology in an educational manner (e.g., Primordial Soup [24] and Strain [25]), but none focused on the human microbiome and most depicted microbes as the villains. We note that while we were designing and testing Gut Check, Susan Perkins at the American Museum of Natural History developed and released a game with very similar themes, called “Gutsy” [26].

In Gut Check, players compete to develop a healthy microbiome for themselves as well as disrupt their opponents’ efforts. The game gives players the ability to play pathogens (i.e., “bad” microbes), beneficial (i.e., “good”) microbes, and numerous opportunistic microbes (microbes that can be “good” or “bad” depending on context). Many concepts from microbiology make an appearance, including antibiotic resistance, lateral gene transfer (the movement of genes from one species to another), hospital-acquired infections, probiotics (microbes with putative health benefits when ingested), prebiotics (substances than can feed beneficial bacteria), and fecal transplants (transplanting a microbiome from one individual to another via feces). A basic game takes around 30 minutes with experienced players, and optimal play involves cycling cards, choosing where to play microbes, timing various events, and deciding whether to foster your own efforts (i.e., build your own microbiome) or interfere with your opponents.

As part of the design and development process, we did playtesting with both gamers (individuals who self-describe as often playing board games) and biologists in order to develop the twin goals of education and player engagement. These different perspectives were invaluable and highlighted the challenge of educational game design. For example, a microbiologist might ask “why does a fecal transplant (a beneficial and relatively benign medical procedure) cause you to lose health?” whereas a gamer would respond “because otherwise the card is too powerful to be balanced (e.g., it makes the game too easy to win)”. Conversely more than a couple of gamers asked about the utility of the lateral gene transfer card, but the application of transferring antibiotic resistance from a beneficial to a pathogenic microbe is intuitive to a microbiologist.

To us, one of the most interesting parts of development was identifying real-life organisms to replace what started out as unnamed placeholders for key microbes. Pathogens were easy to decide on and were mostly chosen for either their reported importance in the human microbiome (e.g., *Clostridium difficile*, responsible for numerous fatal hospital-acquired infections) or their likelihood to be recognized by nonscientists (e.g., *Salmonella)*. However, most of the playtesting was done with the beneficial microbe cards labeled as “Good Bug #2” or “Opportunistic Bug #4” or such. Finding real-world equivalents that had been shown to behave like their in-game counterparts turned out to be challenging. In early versions of the game, there were microbes that allowed a player to digest lactose, grains, or meat. While we were able to find plausible microbes that could carry out the digestion of lactose and grains, we were unable to find solid examples of putative meat digesters. In the final version of Gut Check, this is reflected in several ways: “meat” became “plants,” “Good Bug #2” became *Bacteroides ovatus*, and “Opportunistic Bug #4” became *Treponema carateum*, two bacteria that are putatively involved in digesting fiber.

## Production

**Fig 2:**
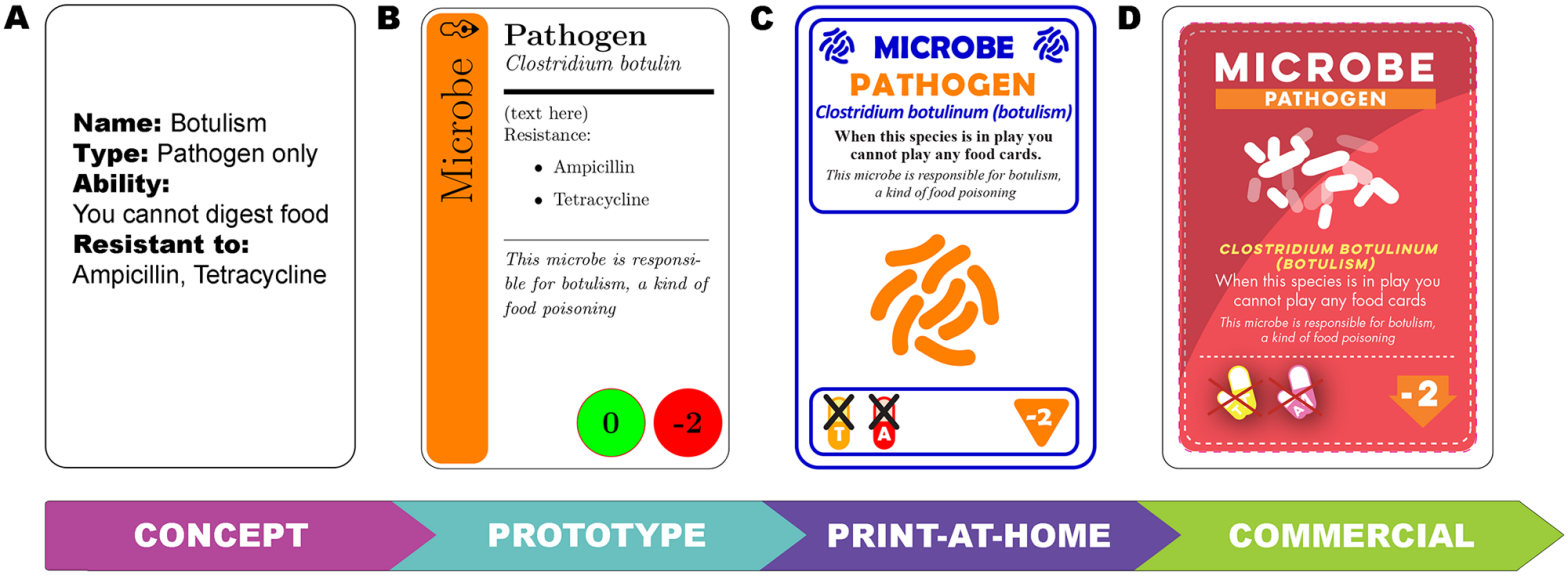
Evolution of a Gut Check card—from concept stage to the commercial version. Throughout the whole process we continuously playtested the game, first for game mechanics, then for card readability, and finally for errors. (A) At the concept stage, “cards” consisted of text in a PowerPoint slide, which were hard to handle when printed. (B) Later, we obtained an open-access card template online that we modified for the prototype version as shown here [28]. This is the step in which the vast majority of playtesting occurred; a script for converting text in an Excel file to the cards greatly facilitated this stage. (C) More playtesting was required for the professional design used in the print-at-home version since we attempted to replace text with icons whenever possible. (D) A final round of testing was required with the new art for the commercial version.

The process of going from the concept of “Gut Check” to a full-blown print-at-home game with professional graphics took us around a year, and it was fairly straightforward to publish the game once complete on our website (Fig 2). Even though we made the game easily available for free online, many players and educators asked us for a commercial version. Some wanted to have a boxed copy of the game to give as a gift, while others were simply not interested in printing and paper-cutting out the cards and would rather pay for a quality product made by someone else. Eager to oblige, we set out to make Gut Check into a more professionally made physical entity and immediately we began to hit a variety of unanticipated snags, specifically production and distribution costs. One option was to get an educational grant, get the game printed, and sell and distribute it ourselves. This had the advantage of being conceptually straightforward but had the disadvantages of having to find the money, become a business (to collect and pay sales tax) and dealing with mailing out games. This does not even count the complications of manufacturing itself such as color matching; box, piece, and card dimensions; and printing scale. Another option to fund a commercial version could have been through Kickstarter, a crowdfunding website, which was suggested to us by many people. However, running a successful Kickstarter campaign is an incredible amount of work (e.g., [27]), and does not solve the problems of sales tax and distribution. A third possibility was to “pitch” the game to an existing game manufacturer and have them produce, sell, and distribute the game. This seemed to be a very reasonable approach, but one that would take a fair bit of legwork and networking and could result in a loss of control over the final product. In our case, we were fortunate to partner with a biotech company, MO BIO Laboratories, a QIAGEN Company, who agreed to undertake developing a professional version of the game into a promotional product. This required new art to “brand” the game, but the mechanics remained unchanged. MO BIO took care of the manufacturing and distribution, a win for everyone concerned at the time. However, when MO BIO was later acquired by QIAGEN, the new parent company opted to no longer sell the game, highlighting the loss of control involved in the corporate sponsorship approach. We are currently pursuing other distribution options to ensure that the game remains publicly available.

## Education

While designing Gut Check, we had in mind a target audience of high school students and early-stage university students interested in biology. However, the game was never explicitly designed for classroom use; instead, we were envisioning use in casual situations for fun. Given that Gut Check has been played by kids as young as six years old and has been used in a number of high school and college classrooms, we clearly underestimated the both target age group and context. We have been gratified and humbled to see Gut Check used in educational settings, both at the high school and college/university levels. To date, no one has conducted a rigorous assessment of learning gains associated with the use of Gut Check in the classroom, but at least anecdotally it has been popular with students. Box 1 shows a few example quotes from students and teachers about their perceptions of the game. These quotes were solicited by contacting a few teachers who had mentioned the use of the game in their classroom on Twitter. One of our near-term goals is to design appropriate assessment to measure any learning gains in the classroom. A casual review of the existing educational literature found a very limited number of studies assessing the use of board games in the classroom, and although the results were positive overall [29,30], many of these studies were inconclusive [31,32], indicating both the difficulties involved in evaluating the benefits of board games on student learning and a need for better methods of assessment.

The learning goals for Gut Check were not couched in relation to standards or classroom curriculum; they were based on aspects of microbiology that we wanted to emphasize. For example, within the game, both antibiotics and opportunistic bacteria can be “good” or “bad” depending on context, a point that we think is not always appreciated by nonmicrobiologists.

**Box 1: Quotes about Gut Check**

“I've found Gut Check to be an incredibly useful way to teach students about their microbiome. In my 9th grade biology classes we used it as a jumping-off point after learning about natural selection and antibiotic resistance. Gut Check allows students to immediately apply their understanding to a real world scenario—themselves! My high school students even came in during lunch to play—and had a great time giving each other botulism!”–Teacher, California

“I found all the different microbes and their effects and resistances to be very interesting. For example, I was really interested by the opportunistic microbes, as I found the fact that they can be either beneficial or harmful depending on how they are used to be very cool. Overall, it was really fascinating to have a fun sort of visual on how these microbes can affect the body.” –9th grade student, California

“My students found Gut Check to be a curiosity at the start of the semester. By the end of the term, my students kept asking if we'd play it again. I knew it was useful when they made connections between the microbes on the cards and what we learned from reading papers”–Professor of a “Human Microbiome” class, Alaska

## Advice to others

We have been asked several times what advice we would give to someone also wanting to develop a fun educational game in a science field. Our suggestions are divided into two sections: design and manufacturing. We hope to see many more challenging and fun educational science games in the future. We feel that it is important to note that none of the authors, or anyone involved in the design or playtesting stages of the game, had any prior experience with game design or production. While such experience would no doubt be incredibly valuable, it is not a prerequisite for success.

Our first design suggestion would be playtest, playtest, playtest. By the time you are done playtesting the game, everyone you know should be completely sick of it and never want to see it again. While this might seem obvious, we have played numerous games (educational or otherwise) where major flaws or problems cropped up in the games that should have been eliminated through playtesting. As discussed above, playtesting should ideally involve people from a variety of backgrounds, including those who are well versed in the science of the game as well as nonscientists who play a lot of board games. We estimate that our many months of playtesting involved at least a dozen scientists, several self-described “gamers” without a scientific background, and several people who were both “gamers” and scientists.

On a related note, prototyping early in the process is really helpful for developing a feel for how the game will actually play. Also, we would encourage people to dive into the extensive resources available for people developing new board games. These resources are not targeted specifically at educational games but are incredibly helpful in helping find ideas about game mechanics including balance, gameplay, probability, time management, etc. Examples include boardgamegeek.com, the Board Game Designers Forum [33], published works [34–36], and blogs such as Game Precipice [37].

When thinking about production and manufacturing, we want to emphasize the amount of work it actually entails to go from an idea at lab meeting to a finished product, available for purchase on a shelf somewhere [38]. We say this not to discourage people from designing a game, but to suggest that they contemplate the whole process from conception through production. As with game design, there are numerous online resources which are incredibly helpful for understanding the production and manufacturing process. One of the most difficult decisions you will face will be economy of scale: printing gets much, much cheaper per unit the more you print. For example, our preliminary quote from Delano Service Inc. for Gut Check was $6.26 per game at 5,000 copies, $7.90 per game at 2,500 copies, $12.83 per game at 1,000 copies, and $19.70 per game at 500 copies. The large decrease in per-unit price going from 1,000 to 2,500 copies comes from a change in printing technology at larger volumes. A large percentage of the total game cost (almost 50% at 500 copies) is the production of game boards, which may be the reason that many educational games consist of cards only. Thus, manufacturing details are intricately linked with game development. For example, to achieve a particular price point per game, compromises might be made in game design (e.g., using wooden blocks instead of custom game pieces) and game mechanics (e.g., number of game pieces used). Thinking about getting from A to Z before even starting can both help along the way and save time later.

Knowing the funding landscape in advance would also be a huge advantage for someone wanting to develop an educational game. For example, obtaining a grant for a set amount of money would constrain the printing and design options from the start. On the other hand, a crowdfunding approach leaves the designers with a lot of up-front work for an unknown amount of future capital. Corporate sponsorship, the route that we took in the end, trades financial security for a lack of control. Were we to ever design another game, we would make this decision in advance of the design process. Other things that we would do differently include ensuring a long-term distribution channel (e.g., fulfillment by Amazon), involving educators in the design process itself, and creating a plan for formal pedagogical assessment before finalizing the game. Lastly, our focus here was primarily on making a fun and challenging game, and we did not adequately consider the educational utility from the perspective of a teacher. Considering things like target audience, classroom setting, length of a high school class, and specific curriculum goals would have lowered the barriers to classroom use.

## Conclusions

Science pedagogy games have the potential to engage and excite students and aid in the retention and learning of knowledge. To be sufficiently compelling, we believe such games should be both fun and challenging. To successfully produce a “fun and challenging” game requires extensive playtesting and a significant investment (time and money) in production and manufacturing. Fortunately, the increasing popularity of such board games, abundant internet resources, and the existence of crowdfunding all help lower the barriers to success. We hope Gut Check can serve as a model of a “fun and challenging” science pedagogy board game, and hope to play many others in the future. Funding organizations wishing to support such development could offer calls explicitly for game development, as well as opportunities to foster collaboration between scientists and game designers.
